# Learning performance is linked to procedural memory consolidation across both sleep and wakefulness

**DOI:** 10.1038/s41598-017-09263-5

**Published:** 2017-08-31

**Authors:** Frida H. Rångtell, Swathy Karamchedu, Peter Andersson, Lieve van Egmond, Tyra Hultgren, Jan-Erik Broman, Jonathan Cedernaes, Christian Benedict

**Affiliations:** Department of Neuroscience, Uppsala University, SE-751 24 Uppsala, Sweden

## Abstract

We investigated whether learning performance in a procedural finger tapping task ﻿﻿before nocturnal sleep﻿﻿ would﻿ predict performance gains after sleep in 60 young adults. Gains were defined as change in correctly tapped digit sequences between learning (12 trials﻿ ad﻿minist﻿er﻿ed in the evening) and retesting (3 ﻿trials﻿ administere﻿d in the morning after sleep). The same task was also administered to a separate wake group (N = 54 young adults), which learned in the morning and was retested in the evening.﻿ Learning performance was determined by either using the average performance on the last three learning trials or the a﻿verage performance on the best three learning trials. Our results demonstrated an inverse association between learning performance and gains in procedural skill, i.e., good learners exhibited smaller performance gains﻿ across ﻿both wakefulness and sleep than poor learners. Regardless of learning performance, gains in finger tapping skills were greater after sleep than daytime wakefulness. Importantly, some of our findings were influenced by how learning performance was estimated. Collectively, these results suggest that learning performance and the method through which it is estimated may influence performance gains in finger tapping skills across both sleep and wakefulness.

## Introduction

Sleep facilitates the storage of newly acquired declarative memories^1^, such as factual knowledge. Sleep also seems to contribute to procedural memory processing (see for instance^2, 3^); however, its role in procedural memory consolidation is less clear^4^. Sleep after training of motor skills, like finger sequence tapping, can produce substantial improvement (i.e., a gain in skill at retesting compared to skill level at learning), pointing towards an active reprocessing of skill representations during post-learning sleep^2, 5^. On the other hand, a recent meta-analysis involving 1,296 subjects found no conclusive evidence that sleep enhances consolidation of newly acquired procedural skills^6^. Further complicating the debate, procedural memory performance boosts also occur after short rest periods without intervening sleep^7–9^, and somatosensory targeted memory reactivation during sleep does not further increase gains in motor memory^10^. This pattern of data makes it unclear whether or not sleep leads to an “offline” enhancement of newly acquired procedural skills.

One factor that likely exerts considerable influence on sleep-dependent consolidation of procedural skills – and could thus be causing variance in results between studies – is baseline learning performance. Pointing in this direction, an improvement in motor skill across sleep was for instance only seen when pre-sleep training was restricted^11^. Furthermore, fast but not slow learners exhibited performance gains across sleep following a procedural oculomotor sequence task^12^. There is also evidence against the view that learning performance before sleep determines gain in procedural skill following sleep. For instance, doubling the intensity of initial training on a finger sequence tapping task did not change the subsequent quantity of overnight sleep-dependent learning^13^.

With the paucity of results in mind, the present analysis investigated whether learning performance in the evening would predict performance gains in procedural skill at delayed retesting scheduled in the morning after nocturnal sleep. For this purpose, we collated finger-tapping data of 60 subjects who had participated in sleep control conditions during previous studies from our laboratory^14–16^ (for a detailed description, see the methods section). In order to examine whether this hypothesized correlation between learning performance and performance gains in finger skill would be specific for sleep, a separate wake group including 54 subjects was administered the same finger-tapping task. The wake group learned in the morning and was retested in the evening.

## Results

### Group characteristics

The sleep (N = 60) and wake groups (N = 54) did not differ in age (22.9 ± 0.3 vs. 23.6 ± 0.4 years, P = 0.16, Mann-Whitney-U test; Cohen’s *d* = 0.27), BMI (22.4 ± 0.2 vs. 22.4 ± 0.3 kg/m^2^, t = −0.16, df = 112, P = 0.87, independent Student’s t test; Cohen’s *d* = 0.03), or in gender ratio (women/men, 17/43 vs. 18/36, Pearson Chi-Square = 0.33, P = 0.56). The polysomnographic analysis revealed that subjects in the sleep group slept in total 435 min ± 4 min in the post-learning night (time spent in sleep stages is shown in Table 1). Self-reports from subjects in the wake group indicated that they spent on average 478 min ± 7 min in bed (in their homes) during the night preceding study participation.

**Table 1:** Summary of sleep variables and composition for the post-learning sleep in the sleep group.

Sleep variable	*Mean*	*SEM*
*SOL (min)*	20.0	1.9
*TST (min)*	435.0	4.1
*WASO (min)*	31.4	3.0
*N1 (% of TST)*	4.1	0.4
*N*2 *(% of TST)*	46.4	0.9
*SWS (% of TST)*	26.6	1.0
*REM (% of TST)*	22.9	0.6

N = 60. Abbreviations: SOL, sleep onset latency; TST, total sleep time; WASO, wake after sleep onset; N1, sleep stage 1; N2, sleep stage 2; SWS; slow-wave sleep; REM, rapid eye movement sleep.

### Procedural memory performance

The computerized finger-tapping memory task requires participants to use their non-dominant hand to repeatedly tap a 5-digit sequence comprised of digits 1–4 (e.g. 4-1-3-2-4), as fast and as accurate as possible during 30-second trials. This task consists of 12 learning trials and 3 retesting trials. In the present study, ﻿the sleep group slept a full night be﻿t﻿ween learning (scheduled in the evening) and retesting (scheduled in the morning). In contrast, in the wake group subjects stayed awake ﻿between learning (scheduled in the morning) and retesting (scheduled in the evening on the same day). Performance gains in finger-tapping skill are usually estimated by dividing averaged scores across the three retesting trials by averaged scores from the final three learning trials. However, it must be noted that averaging scores from the final three learning trials (out of 12 learning trials) may mask a participant’s actual learning performance^17^. With this concern in mind, in the present study, averaged scores from the best three trials during the training period ﻿were used as additional measure of learning performance. All dependent variables, i.e., learning performance, retesting performance, and performance gains between learning and retesting were normally distributed.

A repeated measures ANOVA utilizing time (learning, defined ﻿as average performance on the last three learning trials re-testing, defined a﻿s average performance on the three retesting ﻿trials;﻿ within-subject factor), condition (sleep/wake; between-subject factor), and sex (male/female; between-subject factor) revealed main effects of condition and time on finger skill performance (numbers based on estimated marginal means; sleep vs. wake, 20.1 ± 0.6 vs. 17.6 ± 0.6 correct sequences, P = 0.003; learning vs. re-testing, 17.5 ± 0.4 vs. 20.2 ± 0.4 correct sequences, P < 0.0001). Participants’ sex did not influence their finger skill performance (numbers based on estimated marginal means; men vs. women, 19.2 ± 0.5 vs. 18.5 ± 0.7 correct sequences; P = 0.372). Additionally, there was an interaction between condition and time (P = 0.002). No other interactions were found. Further details about the repeated measures ANOVA can be found in Table 2.

**Table 2:** Overview of analyses setups and results for the ANOVAs.

Analysis			uniANOVA		uniANOVA		uniANOVA		rmANOVA	
Dependent			Learning performance		Retesting performance		%Gain in performance		Performance	
Measure of performance			*Mean best 3 trials*	*Mean last 3 trials*	*Mean best 3 trials*	*Mean last 3 trials*	*Mean best 3 trials*	*Mean last 3 trials*	*Mean best 3 trials*	*Mean last 3 trials*
**Main effects**	*Condition (Sleep/Wake)*	*F*	7.3	4.4	13.9	13.9	3.7	7.2	11.5	9.4
		*P*	**0.008**	**0.039**	**0.0003**	**0.0003**	0.055	**0.008**	**0.001**	**0.003**
		*η* ^2^	0.06	0.04	0.11	0.11	0.03	0.06	0.09	0.08
	*Sex*	*F*	2.2	0.8	0.7	0.7	0.9	<0.1	1.5	0.8
		*P*	0.14	0.385	0.4	0.4	0.333	0.951	0.231	0.372
		*η* ^2^	0.02	<0.01	<0.01	<0.01	<0.01	<0.01	0.01	<0.01
	*Time (Learning/Retesting)*	*F*							21.1	114.8
		*P*							<**0.0001**	<**0.0001**
		*η* ^2^							0.16	0.51
**Interactions**	*Condition × Sex*	*F*	<0.1	0.1	<0.1	<0.1	0.3	0.3	<0.1	<0.1
		*P*	0.767	0.74	0.911	0.911	0.608	0.604	0.834	0.82
		*η* ^2^	<0.01	<0.01	<0.01	<0.01	<0.01	<0.01	<0.01	<0.01
	*Condition × Time*	*F*							4.9	10.2
		*P*							**0.029**	**0.002**
		*η* ^2^							0.04	0.09
	*Time × Sex*	*F*							0.8	<0.1
		*P*							0.379	0.956
		*η* ^2^							<0.01	<0.01
	*Condition × Time × Sex*	*F*							<0.1	0.1
		*P*							0.782	0.72
		*η* ^2^							<0.01	<0.01

N = 114 for all analyses. Abbreviations: F, F-values (1;110); P, P-values (P ≤ 0.05 are indicated in bold); η^2^, Partial Eta Squared; uniANOVA, univariate ANOVA; rmANOVA, repeated measures ANOVA.

**Figure 1:**
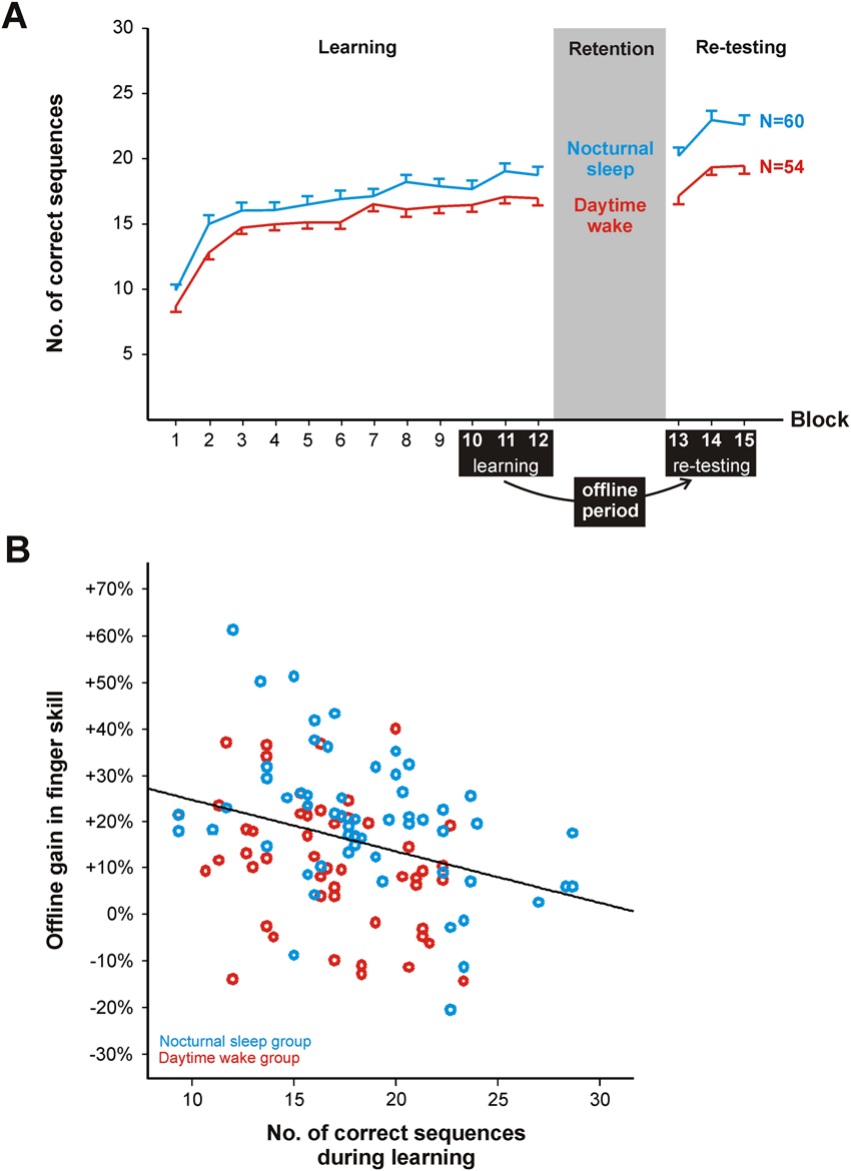
Association between learning performance, type of retention interval (nocturnal sleep vs. daytime wake), and relative offline gain in newly acquired procedural skills. (**A**) Performance in the procedural memory task at learning and retesting, split by sleep (N = 60) and wake (N = 54) groups. Learning performance was defined as the average number of correctly tapped sequences across the final three trials (blocks 10, 11, and 12) during the learning session. Retesting performance was defined as the average number of correctly tapped sequences across the three retesting trials (blocks 13, 14, and 15). Error bars represent SEM. (**B**) Association between learning performance and performance gains (learning performance set to 0%) in finger tapping skill. Note that no interaction between learning performance, sex and condition was found.

Learning performance, defined ﻿as average performance on the last three learning trials, was different between the sleep and wake groups (univariate ANOVA; for details see Fig. 1A, Table 2 and 3). Sleep subjects tapped 1.7 ± 0.8 more correct sequences (based on estimated marginal means) during the final three learning trials than those of the wake group (P = 0.039). At retesting, subjects of the sleep group tapped 3.3 ± 0.9 more correct sequences than those of the wake group (based on estimated marginal means; P = 0.0003; univariate ANOVA; Fig. 1A, Tables 2 and 3). The repeated measures ANOVA, as well as the two univariate ANOVAs for learning and retesting performance, yielded similar results independent of how ﻿learning performance was estimated (that is, either using the average performance on the last three or the average performance on the best three learning trials; see Table 2).

**Table 3:** Descriptive overview of subjects' performance in t﻿he﻿ finger tapping memory task, split by sleep (N = 60) and wake (N = 54) groups. Performance gains in finger skill were calculated by dividing averaged scores ﻿across the three retesting trials by learning performanc﻿e (i.e. ((re-testing/learning)-1) * 100). Learning performance was estimated ﻿by either using the average from the last three learning trials or the average from the best three learning trials (out of 12 learning trials).

Measure of performance	Sleep group						Wake group					
	*Learning*		*Retesting*		*%Gain*		*Learning*		*Retesting*		*%Gain*	
	*Mean*	*SEM*	*Mean*	*SEM*	*Mean*	*SEM*	*Mean*	*SEM*	*Mean*	*SEM*	*Mean*	*SEM*
*Mean best 3 learning trials*	20.29	0.55	21.93	0.61	8.69	1.81	18.15	0.48	18.65	0.52	3.19	1.58
*Mean last 3 learning trials*	18.49	0.57	21.93	0.61	20.10	1.92	16.85	0.48	18.65	0.52	11.64	1.86

Baseline l﻿e﻿arni﻿n﻿g performance differed between the sleep and wake groups (see above). Thus, a univariate ANOVA utilizing between-subject factors sex and condition (sleep/wake) was perf﻿ormed in order to inves﻿tigate whether performance gains in finger skill between learning and retesting would differ between ﻿experimental groups﻿. This analysis revealed that nocturnal sleep conferred greater performance gains in finger-tapping skills than daytime wakefulness (sleep vs. wake: 19.7 ± 2.1% vs. 11.9 ± 2.1%, numbers based on estimated marginal means; P = 0.008; Tables 2 and 3). In contrast, when instead using the average scores from the best three trials during the training period, performance gains in finger skill did not statistically differ between experimental groups (P = 0.055; Table 2). There were no main effects of the participants’ sex in any of these analyses, nor were there any interactions between condition and sex (Table 2).

The performance gains in finger skill across sleep was not correlated with any of the measured sleep characteristics (N = 60; performance gain in finger skill﻿ between learning and retesting as dependent and the following sleep parameters as independent factors): minutes of sleep onset latency (mean of last three: Spearman’s rho = 0.20, P = 0.126; mean of best three: Spearman’s rho = −0.04, P = 0.737), minutes of total sleep time (mean of last three: Spearman’s rho = −0.15, P = 0.257; mean of best three: Spearman’s rho = −0.03, P = 0.806), time in the sleep stages (as percentage of total sleep time) N1% (mean of last three: Spearman’s rho = 0.12, P = 0.359; mean of best three: Spearman’s rho = 0.25, P = 0.057), N2% (mean of last three: Spearman’s rho = −0.12, P = 0.350; mean of best three: Spearman’s rho = −0.17, P = 0.186), slow-wave sleep% (mean of last three: Spearman’s rho = 0.12, P = 0.361; mean of best three: Spearman’s rho = 0.03, P = 0.823) and rapid eye movement sleep% (mean of last three: Spearman’s rho = −0.07, P = 0.607; mean of best three: Spearman’s rho = −0.005, P = 0.967).

In order to examine whether learning performance would﻿ predict performance gains in finger tapping skill between learning and retesting, a linear regression (N = 114) was performed, including the offline gain between learning and retesting as dependent variable, and learning performance as predictor. Moreover, sex and condition (sleep/wake) were utilized as covariates. The analysis revealed that learning performance, defined ﻿as average performance on the last three learning trials, had a major influence on subsequent performance gains in finger skill between learning and retesting (t = −4.42; P = 0.00002; Standardized beta coefficient = −0.38; Fig. 1B). That is, an increase in learning performance by one correctly tapped finger sequence lowered the performance gain by −1.4 ± 0.3%. Similar findings were obtained when using the mean of the best three learning trials as measure of learning performance (mean of the best three trials, t = −2.02; P = 0.045; Standardized beta coefficient = −0.19). No interactions were found (P ≥ 0.359 for all interactions derived from two full-factorial regressions, one for last three trials and one for best trial).

The data from the sleep group was derived from four separate in-lab studies from our group. Thus, the timing of learning and length of offline interval varied among subgroups of the sleep group (see methods section). A univariate ANOVA (N = 60) demonstrated that performance at learning did not vary between subgroups (between-subject﻿﻿ factor), irrespective of how performance was measured (last three trials: F(3;56) = 0.55, P = 0.651, Partial Eta Squared = 0.03; best three trials: F(3;56) = 1.84, P = 0.151, Partial Eta Squared = 0.09). This suggests that timing of learning and other differences between the sleep studies did not have a major influence on learning performance. In addition, performance at retesting did not vary between sleep subgroups (univariate ANOVA; N = 60; last and best three trials: F(3;56) = 0.63, P = 0.596, Partial Eta Squared = 0.03). When estimating performance gains ﻿in finger sk﻿ill by dividing averaged scores across the three retesting trials by averaged scores from the last three (or from the three best) learning trials, gains in finger skill did not differ between studies (univariate ANOVA; N = 60; last three trials: F(3;56) = 0.70, P = 0.553, Partial Eta Squared = 0.04; best three trials: F(3;56) = 2.30, P = 0.087, Partial Eta Squared = 0.11). Length of offline interval (ordinally scaled) did neither influence performance at retesting (Spearman’s rho = −0.01, P = 0.95), n﻿or performance gains in procedural skill between learning and retesting when averages from the last three learning trials were used for quantifying sleep-dependent procedural memory consolidation (Spearman’s rho = 0.17, P = 0.21; N = 60). On the other hand, when averages from the best three learning trials were used for calculating offline gains in finger skill, there was a correlation between gains in procedural skill and length of offline interval (N = 60; Spearman’s rho = 0.31, P = 0.015).

## Discussion

In the present study, we found that baseline learning performance affects how well a newly acquired procedural skill will be consolidated. Participants with high learning performance exhibited less pronounced consolidation benefits during nocturnal sleep or daytime wakefulness, as compared to those with low baseline performance. However, our study also raises an important methodological issue. Performance gains in finger-tapping skill were greater after sleep than wakefulness when performance gains in finger skill were estimated by dividing averaged scores across the three retesting trials by averaged scores from the last three learning trials. No such differences between sleep and wakefulness were found when gains in finger-tapping skill were instead estimated by dividing the average across the three retesting trials by the average from the three best learning trials. Hence, not only learning performance but also the method through which it is estimated may determine the influence of sleep and wakefulness on the consolidation of newly acquired procedural skills.

Our main result was that learning performance was inversely linked to post-learning gains in finger-tapping skill, irrespective of the type of retention interval (i.e., nocturnal sleep or daytime wakefulness). Hippocampus and striatum, as well as their interactions with cortical structures, play important roles in procedural sequence learning^18^. It has been proposed^18^ that initial procedural learning involves hippocampal-cortical activity, and learning-associated hippocampal activation to be important for subsequent sleep-dependent offline processing of the procedural sequence. A shift from the initially increased hippocampal-cortical activation toward striato-cortical activation would instead relate to an increased automatization when performing the motor sequence. This shift would also render the newly acquired memory more robust and less prone to interference; thus, the striato-cortical activation is rather linked to maintenance of procedural skill performance, than to enhancement. A person with a high procedural ability, compared to someone with a lower, might then be more likely to have undergone a greater shift from hippocampal-cortical activation to striato-cortical automatization during learning. The memory trace could therefore be more robust and benefit less from subsequent offline processing.

Sleep in the present study conferred greater consolidation benefits of procedural skills than daytime wakefulness, which is in line with some but not all previous observations^1–6, 19, 20^. Specifically, time in sleep stage 2 has been implicated as important for procedural memory processing during sleep^3, 21^. However, in line with our findings, some studies have not shown correlations between sleep stages and performance gain in procedural skills^17, 22^. The question is how sleep compared with wakefulness may improve the consolidation of newly acquired procedural memories. It has for instance been suggested that during initial learning of a longer procedural sequence, the sequence is encoded in smaller subsequences – or “chunks” – each of which will be processed as single memory units^23, 24^. This “chunking” would facilitate learning of longer sequences that otherwise would be very demanding. Sleep seems to promote merging of the chunked sequences into a single memory representation. Kuriyama *et al*.^25^ for instance found that within-sequence transition speeds during a finger-tapping task were changed during sleep so that the high degree of “chunking” that was seen during learning, was evened out by sleep, and post-sleep performance therefore exhibited a smoother performance.

There are some points to consider when interpreting the results of our study. Subjects in the sleep group performed better at learning (i.e., they tapped more correct finger sequences) than those of the wake group, despite both groups being comparable with respect to age, BMI, and educational status. It is possible that the difference in learning performance between the sleep and wake groups (learning session scheduled in the evening and morning, respectively) is a result of circadian variations in learning capacity^26, 27^. Despite a higher sleep pressure in the evening due to longer time awake, if evening learning occurs within the wake maintenance zone – a time window of reduced sleep propensity occurring immediately before the steep evening increase in the sleep-promoting hormone melatonin – performance might actually be enhanced^28^. This could explain why the sleep group performed better at learning compared to the wake group in our study. Other factors that could also account for the difference at learning between the sleep and wake groups are sleep duration and quality in the night preceding the experimental day. We tried to minimize the potential confound of both between-group and inter-individual differences in learning on memory consolidation. This was achieved through dividing post-retention recall performance by learning performance. However, it cannot be ruled out that differences at learning between the sleep and wake groups may partially explain the observed difference in memory consolidation between the experimental groups.

Going further, the gender groups were not perfectly balanced as fewer women than men were included in the study. In addition, all included women took oral contraceptives to minimize the confounding effects of variations in sex hormones across the menstrual cycle on consolidation of procedural memories^29^. Thus, observed findings might be different in freely cycling women. Finally, when utiliz﻿i﻿ng the average performance on the last three learning trials (out of 12 learning trials) to estimate individual performance gains in finger skill between learning and retesting, we found that sleep conferred greater consolidation benefits than daytime wakefulness. In contrast, no such difference was found when utilizi﻿ng the average performance on the best three learning trials to estimate individual performance gains in finger skill between learning and retesting. Thus, it must be kept in mind that the choice of the method that is used to estimate learning performance could partially ﻿account for differences in the consolidation of finger tapping skills between sleep and wakefulness.

## Conclusions

Our results suggest that both learning performance and the method through which it is estimated may determine the magnitude of subsequent procedural memory consolidation following both sleep and wakefulness. These factors could therefore account for between-study variance in procedural offline gains observed in previous studies^6^.

## Material and Methods

### Participants

A total number of 114 healthy adults (age range 18–30 years; 31% women) were included in the analysis of the present study. All women took oral monophasic contraceptives containing progesterone and estrogen at the time of the study, but were otherwise free of medication; none of the men were on any kind of medication. Note that experiments of the female subjects were not scheduled during their menses. Prior to study inclusion, screening questionnaires were administered to recruit participants with normal sleep habits (sleep duration between 7–9 hours per night, self-reported sleep onset time between ~10 pm to 12 pm) and general good health status. All participants provided written informed consent, and the studies included in the analyses were conducted according to the Declaration of Helsinki and approved by the Regional Ethical Review Board in Uppsala, Sweden.

### Sleep group

Data from the sleep group (N = 60) was derived from four separate in-lab studies from our group (unpublished and published, i.e., Rångtell *et al*., investigating effects of LED screen light on sleep^14^; Cedernaes *et al*., investigating how short sleep affects memory consolidation^15^ and Cedernaes *et al*., investigating how stress after sleep loss impacts memory functions^16^). Note that this merged data has not been published elsewhere, nor did any of these studies investigate whether learning performance would link to subsequent offline gain in procedural skill. Across all sleep studies, subjects spent an adaptation night in the sleep laboratory prior to experimental nights. This was to reduce possible bias from the first-night effect on sleep maintenance and quality in the experimental sleep night^30^. In addition, before the test night, participants were instructed to go to bed and wake up at regular times (i.e. between 2100–2400 hrs and 0600–0900 hrs, respectively). In a brief verbal interview at arrival on the testing day, all subjects confirmed that their pre-baseline sleep was as regular as possible with respect to length (i.e., 7–9 hours), quality, and timing.

Across studies, subjects arrived at the laboratory in the afternoon or early evening. Between 8 pm and 10 pm, participants were administered the procedural memory task (description of the task can be found below). Eleven of the subjects learned at 8 pm, twenty-four at 9 pm, thirteen at 930 pm, and twelve at 10 pm. During the night following learning, participants slept in our sleep laboratory at Uppsala University. Lights were switched off either around 1030 pm (N = 25) or 11 pm (N = 35). The next morning, lights were switched on either around 630 am (N = 24) or 7 am (N = 36), resulting in a sleep opportunity between 8 hrs (N = 47) and 8.5 hrs (N = 13). Retesting of procedural memory was scheduled ~1 hr after awakening.

Polysomnography (including electroencephalography [EEG], electrooculography [EOG], and electromyography [EMG]) was continuously recorded with Embla A10 recorders (Flaga hf, Reykjavik, Iceland). The EEG montages varied between the sleep studies: Fp2, C3; *or* Fp1, Fp2, C3, C4; *or* F3, F4, C4, O1, O2; *or* F3, F4, Cz, O1, O2, referenced to the contralateral mastoids. Sampling rates varied from 100 to 200 Hz. Polysomnography was analyzed according to standard criteria^31^.

### Daytime wake group

Following a night of sleep at home (i.e. subjects were instructed to go to bed 12 pm at the latest and to sleep between ~7–9 hours), subjects (N = 54) arrived at the laboratory at Uppsala University between 8 am and 10 am. First, subjects filled in a sleep-diary to document the duration of sleep in the previous night. They were then administered the learning version of the procedural memory task (described below). Eight hours later (similar in duration to the sleep opportunity for our sleep group), they returned to the lab for retesting. Between learning and re-testing (i.e. during the wake offline interval), subjects engaged in everyday activities. They were however instructed not to exercise extensively, avoid drinking caffeinated beverages, stay awake (i.e. no naps were allowed), and not to practice the newly learned finger sequence. Subjects’ compliance with our experimental protocol was ensured by a questionnaire administered before the retesting session was started.

### Procedural memory task

This computerized task can be used to measure consolidation of procedural memory^32–34^. Briefly, the non-dominant hand is used to repeatedly tap a 5-digit sequence comprised of digits 1–4 (e.g. 4-1-3-2-4), as fast and accurately as possible for 30 seconds per trial. During learning, this sequence is tapped in twelve subsequent trials. After each trial, there is a 30-second long break. At retesting, subjects repeat the procedure in three trials. The mean performance (correct number of sequences per trial) of the final three learning trials (i.e. trial 10, 11, and 12) and the mean performance across the three re-testing trials (i.e. trial 13, 14, and 15) was used to calculate offline gain (i.e. ((re-testing/learning)-1) * 100), unless otherwise specified.

### Statistical analysis

Data are shown as mean ± SEM, unless otherwise stated. Normal distribution was visually assessed, as well as analyzed using the Shapiro-Wilk test. Learning performance in the finger-tapping task was defined in two ways (mean of the final three learning trials and mean of the three best learning trials), and all statistical evaluations were applied to both of these data sets.

Data from subjects whose performance (defined as the average of the final three trials) at learning or re-testing, or offline gain from learning to retesting differed from the group mean (sleep and wake group, separately) by more than two standard deviations were identified as outliers and excluded from analysis (N = 13, i.e. the initial sample consisted of 127 subjects). This criterion applied to seven subjects of the sleep group and six subjects of the wake group.

A repeated measures ANOVA was used to test if the performance would differ between the between-subjects factors condition (sleep/wake) and sex, as well as for the within-subject factor time (learning/re-testing). In addition, three univariate ANOVAs were used to test whether learning and retesting performance as well as the gain would vary with condition and sex.

Spearman correlations were used to investigate if overnight gain in finger tapping skills would correlate with sleep variables, such as amount of sleep stage 2 (percentage of total sleep time).

A linear regression model was utilized to analyze possible associations between learning performance (predictor) and performance gains in finger skill between learning and retesting (dependent variable), while controlling for sex and condition. An additional linear regression model investigated possible interactions using a full-factorial design including the interaction terms built by study condition (dummy coded), sex (dummy coded) and learning performance (entered as z-values).

Since the data in the sleep group was derived from four separate in-lab studies, we wanted to check that there were no subgroup differences within the sleep group. Therefore, univariate ANOVAs were performed for performance at learning and retesting, as well as the offline gain, using *sleep study* as between-subjects factor. In addition, Spearman correlations tested whether the length of the offline interval (ordinally scaled) in the sleep group would correlate with performance at retesting as well as offline gain. Overall, the significance level was set to P < 0.05, two-tailed.
